# Nature Notes—VIII
*The previous articles in this series appeared in The Hospital of Nov. 20, 1909, p. 210; Dec. 4, 1909, p. 272; Jan. 8, 1910, p. 423; Feb. 12, p. 571; March 12, p. 693; June 25, p. 384; the last time being on August 13, p. 587.


**Published:** 1910-12-24

**Authors:** 


### NATURE NOTES—VIII.*

Nightjars—A Peculiar Note—White Owls—The Squire's Views—House-martins Building—Swallows in Pursuit—Silence and Moulting.

On summer evenings I derive much pleasure from watching the night-jars in my paddock. Many of these birds spend spring and summer in the covers and borders round my house. I hear them "churring" in the woods after sunset during May and June. The word "churr" nearly reproduces the sound they utter, which is loud and difficult to place. I do not remember to have heard the bird "churr" upon the wing. I think that they utter their song, if song it may be called, when on the ground.

The night-jar is widespread throughout the downland and fairly common, though but little known because so entirely nocturnal in its habits and so essentially a bird of wild places. It loves oak and hazel coppices and wild heaths. Years ago I used to spend summer evenings on a Wiltshire farm that lay on the uplands near a great gorse. By the farm and gorse there is a wide grass lane bordered by an oak and hazel and brambles. This lane was a chosen haunt. Any evening four or five or more of these graceful birds could be watched chasing insects round the oaks. When in pursuit of their prey they sometimes make a clapping sound. They produce this sound, I think, by striking the tips of their wings together above their backs. The flight is peculiarly light and graceful. It is often described as swallow-like, but to my mind that word does not convey an accurate impression. A swallow gives great impression of power and strength in its flight, for it is a compact bird, whilst the light, swift, graceful, but often fluttering flight of the night-jar more resembles the flight of the moths on which they feed. Moths and beetles taken on the wing are the main of their food. In my paddock they hawk around the horses grazing and over a manure heap. The horses, no doubt, unconsciously assist them in their hunting by putting up insects from the grass, and I suppose there is a constant hatching of beetles and such-like insects from the manure. I often see one of them fly over a chestnut tree that is beyond my borders and take a turn round it, but I infer that the chestnut does not yield much food because I never see the birds stay by it for a length of time.

Two white owls frequent the precincts of my house to beat along some high thorn hedges and some rough grass that is the home of a number of field voles and mice. Often they sit together in the chestnut tree to rest from hunting, and make weird hissing noises there just outside my bedroom window. Sometimes I hear the cock-pheasants call indignantly in the corn as the owl passes over them, though they can have no grounds for fear for themselves; perhaps an owl may now and again take a chick. A keeper in the vale told me that the owls do not often do harm by taking young pheasants themselves; his complaint against them was that, as they go about the trees they frighten the young pheasants and send them to the ground, where the foxes pick them up.

Our squire will not allow hand-reared pheasants. The hens lay in the covers and hedgerows and have to take their chance. The number so reared is not, of course, so great as could be put down from pens, but the birds are really wild birds. The squire believes that, since the most careful and most wary mothers will survive and send forth the greatest number of young, natural selection will stock his broad acres with a race fitted to fend for themselves. I believe he is right. Such a race will not chance a fox to avoid a harmless owl.

Some pairs of house-martins have nested on my house. They were late in starting their nests and took long over the building of them; moreover, for reasons I could not fathom, they made several false starts and abandoned the nests they began. On first arriving from the south a little flock of them spent some days examining the eaves, chattering pleasantly as they flew about or clung to the walls. Then in a friendly little flock they spent a short while each day collecting mud from a damp place beneath my paddock gate where the passing in and out of carts has made a depression that holds a puddle and remains muddy after the road is dry. All round the puddle were innumerable prints of tiny feet and little pits from which little beakfuls had been lifted. The birds used to flutter over the puddle and chatter together in a pretty manner and alight upon the mud and fill their beaks with it. A sufficient beakful collected, they would fly up to the selected site of the nest and stick little pellets of mud to the wall. With instinctive geometrical knowledge they kept the rising walls in a segment of a circle, sufficient accuracy being attained by the simple device of clinging always to one spot on the wall and stretching out and working round a centre at their feet to the end of a radius the length of the distance from feet to beak. They use their chins to smooth off their work as a bricklayer uses his trowel. They worked in leisurely fashion, doing but little each day, so the nests grew slowly. In two cases, when the nest was nearly finished, dishonest sparrows took possession, and, with strong beaks projected through the entrance, defied and drove off the rightful owners, and, after giving the nest a slight lining of hay and feathers, laid therein. This has not been a good year for the swallow tribes, I fear. They seem to me to be in small numbers.

During the most of July and August the birds moult and cease to sing. The last birds I heard sing this year were two larks, and they sang under unusual circumstances. It was one evening early in July, half an hour after dark. The moon was bright, though less than full. I was strolling up the road before my house, and suddenly two larks, one on either hand, burst into song. They sang full song as they do by day, though it was so dark I could not see them; neither as they rose nor as they came down again. I was surprised, for I do not remember hearing larks sing by night previously.

---

* The previous articles in this series appeared in The Hospital of Nov. 20, 1909, p. 210; Dec. 4, 1909, p. 272; Jan. 8, 1910, p. 423; Feb. 12, p. 571; March 12, p. 693; June 25, p. 384; the last time being on August 13, p. 587.

